# Supplementary material for: Human INCL fibroblasts display abnormal mitochondrial and lysosomal networks and heightened susceptibility to ROS-induced cell death
Source: PLoS One. 2021 Feb 9;16(2):e0239689. doi: 10.1371/journal.pone.0239689 (PMC7872282; doi:10.1371/journal.pone.0239689)
Supplement: S1 File — (DOCX) [file pone.0239689.s001.docx]

S1 File.

The following fibroblast cell lines were obtained from the Coriell Institute for Medical Research ([www.coriell.org](http://www.coriell.org)):

(1) INCL patient fibroblast cell line GM20389 was derived from the skin of an INCL male patient donor bearing a compound heterozygotic mutation where one allele has a T to C alteration at position 739 in exon 8 of the PPT1 gene (this resulted in a Tyr247His mutation). The other allele has a G to A alteration at position 3 in exon 1 of the PPT1 gene (this resulted in a Met1Ile mutation). The patient cell lines displayed decreased PPT1 enzyme activity (Supplementary Fig. S1).

(2) Wild type human fibroblast cell line GM05659 deriving from an apparently healthy male donor was used as a control.
