# Supplementary figures and images for: Human INCL fibroblasts display abnormal mitochondrial and lysosomal networks and heightened susceptibility to ROS-induced cell death
Source: PLoS One. 2021 Feb 9;16(2):e0239689. doi: 10.1371/journal.pone.0239689 (PMC7872282; doi:10.1371/journal.pone.0239689)

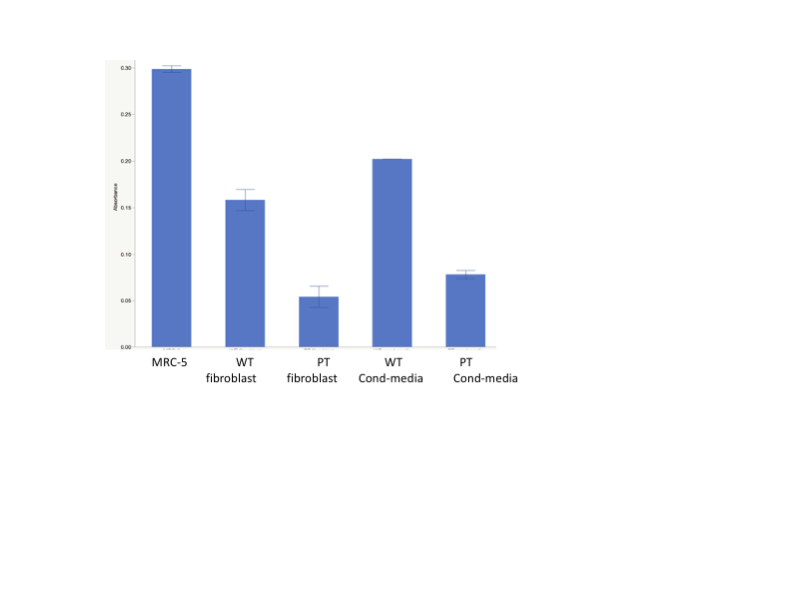

Supplement: S1 Fig — All samples were performed in duplicates. Mean and standard deviation were calculated with P<0.001. (TIF) [file pone.0239689.s002.tif]

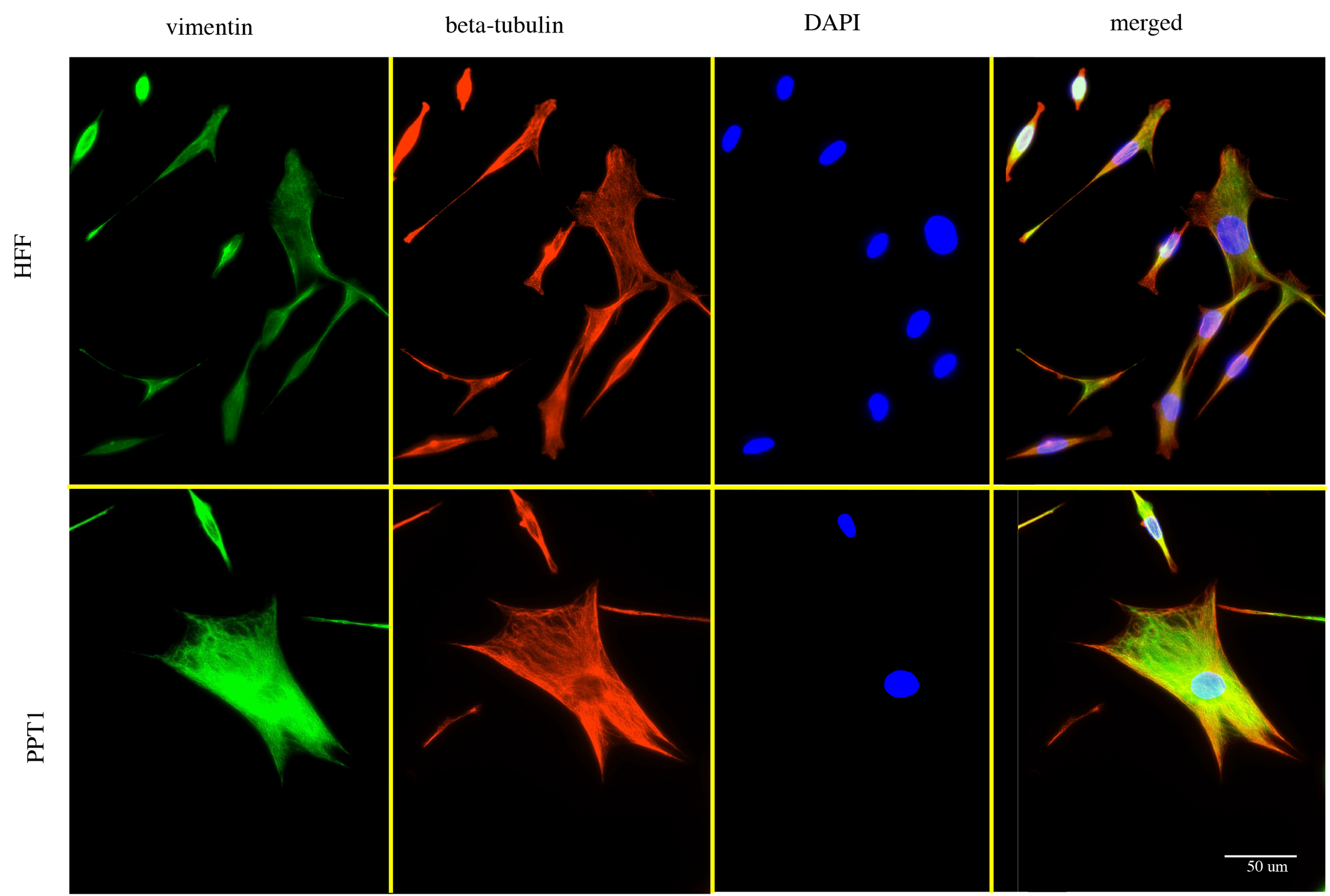

Supplement: S2 Fig — HFF (top row) and PPT1 deficient (bottom row) fibroblasts were probed for vimentin (green), beta-tubulin (red) and counterstained with DAPI (blue). There were no differences observed in the vimentin or beta-tubulin distribution of PPT1-deficient and normal fibroblast cells (merged). (TIF) [file pone.0239689.s003.tif]

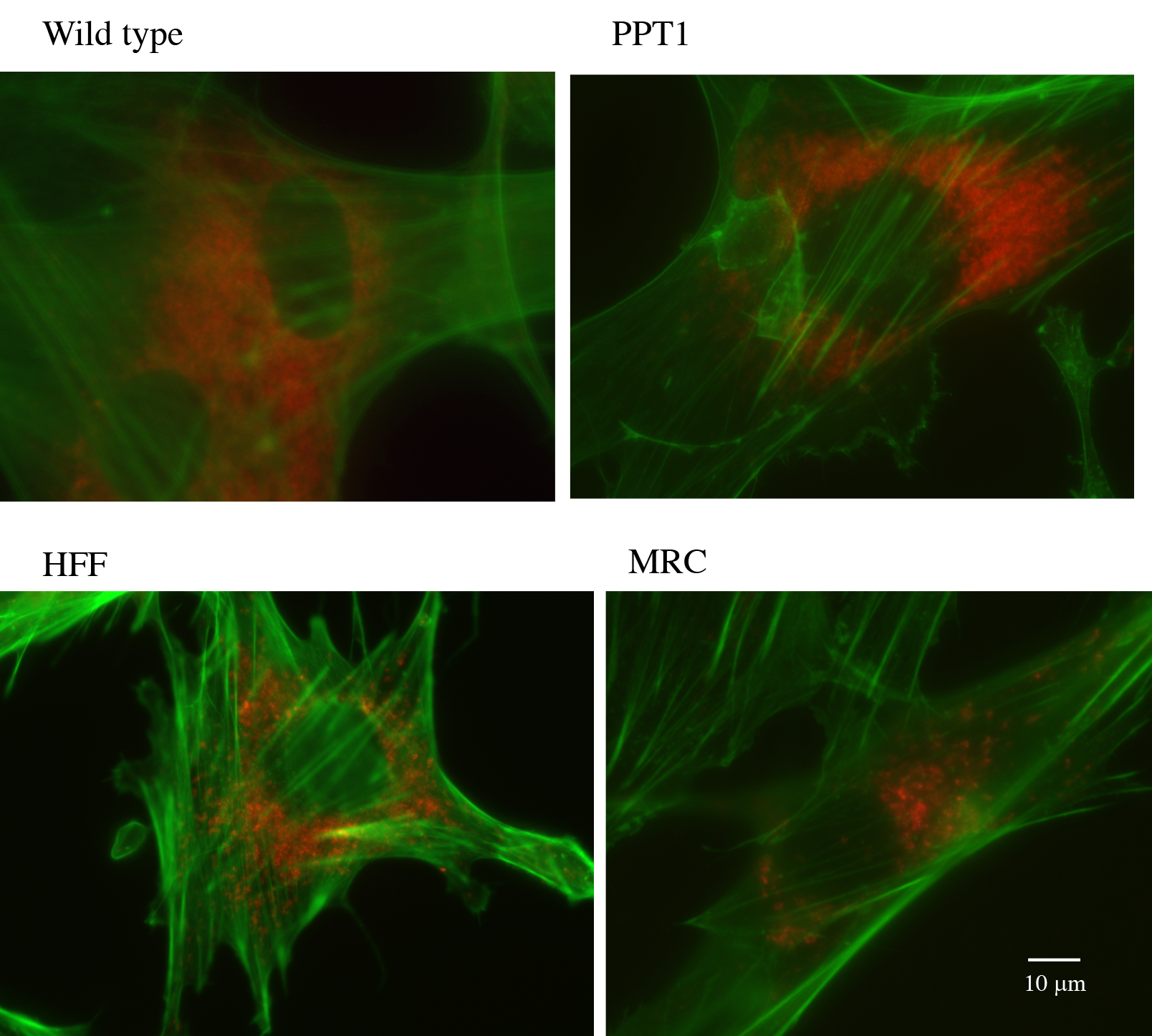

Supplement: S3 Fig — Cells were stained with LAMP1 (red) and Phalloidin (green). (TIF) [file pone.0239689.s004.tif]
